# A Radar Signal Recognition Approach via IIF-Net Deep Learning Models

**DOI:** 10.1155/2020/8858588

**Published:** 2020-08-28

**Authors:** Ji Li, Huiqiang Zhang, Jianping Ou, Wei Wang

**Affiliations:** ^1^School of Computer and Communication Engineering, Changsha University of Science and Technology, Changsha 410114, China; ^2^ATR Key Laboratory, National University of Defense Technology, Changsha 410073, China

## Abstract

In the increasingly complex electromagnetic environment of modern battlefields, how to quickly and accurately identify radar signals is a hotspot in the field of electronic countermeasures. In this paper, USRP N210, USRP-LW N210, and other general software radio peripherals are used to simulate the transmitting and receiving process of radar signals, and a total of 8 radar signals, namely, Barker, Frank, chaotic, P1, P2, P3, P4, and OFDM, are produced. The signal obtains time-frequency images (TFIs) through the Choi–Williams distribution function (CWD). According to the characteristics of the radar signal TFI, a global feature balance extraction module (GFBE) is designed. Then, a new IIF-Net convolutional neural network with fewer network parameters and less computation cost has been proposed. The signal-to-noise ratio (SNR) range is −10 to 6 dB in the experiments. The experiments show that when the SNR is higher than −2 dB, the signal recognition rate of IIF-Net is as high as 99.74%, and the signal recognition accuracy is still 92.36% when the SNR is −10 dB. Compared with other methods, IIF-Net has higher recognition rate and better robustness under low SNR.

## 1. Introduction

Radar signal recognition is a key technology in the field of radar electronic countermeasures. When receiving a radar signal, it is crucial to demodulate the signal to obtain useful information, and how to identify the signal type is the key. The accuracy of signal recognition in a complex electromagnetic environment determines the pros and cons of electronic reconnaissance systems. Due to the emergence of complex electromagnetic environments and various new system radars in modern warfare, electronic reconnaissance and electronic countermeasure systems have brought serious challenges. How to identify the type of radar signal more quickly and accurately is the key and difficult point of radar signal recognition technology.

Traditional radar signal recognition technologies include support vector machine learning (SVM) and traditional five-parameter feature matching algorithm. Li and Ying [1] achieved the purpose of identifying and classifying radar signals by extracting different entropy features. Ying and Xing [2] proposed an improved semisupervised SVM algorithm for radar signal recognition which has high accuracy. Li et al. [3] proposed a deep joint learning method, including deep representation and low-dimensional discrimination, to enhance feature stability and environmental adaptability. The approach achieved a high recognition rate for multiple radar signals under low SNR. Li [4] proposed an SKLEARN system based on automatic machine learning. Through the automatic solution algorithm of the SKLEARN system and the optimization of hyperparameters, the accuracy of radar signal recognition is improved and the stability is more reliable. Feng B et al. [5] proposed a manifold method to reduce dimensionality in high dimensions, extract features, and set an appropriate threshold as a classifier. This method had good accuracy, but did not have good generalization performance. Guo et al. [6] proposed a frequency domain analysis method and an identification method based on the Fast Correlation-based Filter Solution (FCBF) and adaboosting (AdaBoost). Under low SNR conditions, this method is more efficient than manually extracting features for classification. Zhang et al. [7] proposed a machine learning method based on Tree-based Pipeline Optimization Too (TPOT) and Local Interpretable Model-agnostic Explanations (LIME) and used genetic algorithms to optimize the pipeline structure and related parameters. This method can not only optimize the machine learning process for different data sets but also determine the type of radar signal according to the interpretability of the radar signal when there are indistinguishable radar signals in the dataset.

However, traditional radar signal recognition technology requires artificial design of more complex features extraction algorithms and classifiers, which are more difficult to implement and have poor generalization performance. With the development of artificial intelligence (AI), the application fields of deep learning are getting wider and wider. In the field of image recognition, Convolutional Neural Networks (CNNs) is a hotspot in many researches. Its network has ability to represent learning, that is, it can extract high-order features from input information, and can respond to the translation of input features. Denaturation, which can identify similar features in different positions in space, is widely used in computer visualization, natural language processing, and other fields. Qu et al. [8] proposed a multilabel classification network based on the Deep Q-learning Network (DQN), which can be recognized under low SNR. Through the radar signal preprocessing and feature extraction of the convolutional neural network, the network can identify random overlapping radar signals under low SNR. Cai et al. [9] proposed a radar signal modulation and recognition algorithm based on an improved CNN model. In this model, a dense connection block layer and a global pooling layer were added to identify 8 radar signals. Limin et al. [10] proposed a radar signal recognition method based on an improved AlexNet model. At low SNR, they performed smooth pseudo-Wingner time-frequency analysis on a variety of signals using an improved AlexNet model, resulting in a high overall recognition rate.

In this paper, USRP N210 and USRP-LW N210 Universal Software Radio Peripheral (Universal Software Radio Peripheral) are used to simulate the radar signal transmission and reception process, and a total of 8 classes of radar signals, namely, Barker, Frank, chaotic, P1, P2, P3, P4, and OFDM, are produced with the SNR between −10∼6 dB. Then, all classes of signals were distributed through the Choi–Williams distribution function (CWD) transformation to generate two-dimensional time-frequency images (TFIs). As the TFI information location distribution of different radar signals is quite different, some signal information is concentrated in the central area, and some signal information is distributed at the edge. Aiming at the abovementioned problems, this paper designed a global feature balance extraction module (GFBE) and a new IIF-Net convolutional neural network structure which has strong recognition ability for radar signals. By improving the classifier, IIF-Net has reduced the number of parameters and computation and has better identification accuracy and reliability.

## 2. GFBE Module and IIF-Nets

### 2.1. GFBE Module

The traditional radar signal recognition method is based on the conventional 5 parameters: carrier frequency (RF), angle of arrival (DOA), pulse arrival time (TOA), pulse amplitude (PA), and pulse width (PW). However, most of the signal parameters are external features, which are easy to be interfered by the external environment. The external interference will cause the distortion and loss of the signal and reduce the recognition accuracy. CNNs can adaptively learn image features for recognition, which can improve the accuracy of radar signal recognition.

With the development of computer hardware, CNN is widely used in various fields. In the article of the development of convolutional neural network and its application in image classification, Wang et al. [11] analyzed the application and development of CNN in detail. In 2012, Hinton and Alex Krizhevsky proposed AlexNet [12] and successfully applied ReLU [13], Dropout [14], and LRN [13] in CNN for the first time. Visual geometry group networks (VGG-Nets) [15] proposed a 3×3 small convolution filter, which deepened the network to 19 layers. With the increase of the network depth, the problem of network degradation appeared. After enough training times, the accuracy rate on the training set will be saturated or even decreased, and the problem of gradient and information disappearance also hinders the increase of the network depth. Residual net (ResNet) [16] solved this problem by using short skip connection and continued to increase the network depth. In image recognition, in order to extract features better, the image can be reconstructed with super resolution [17]. The improved lightweight network [18] also achieves a good classification effect.

**Figure 1:**
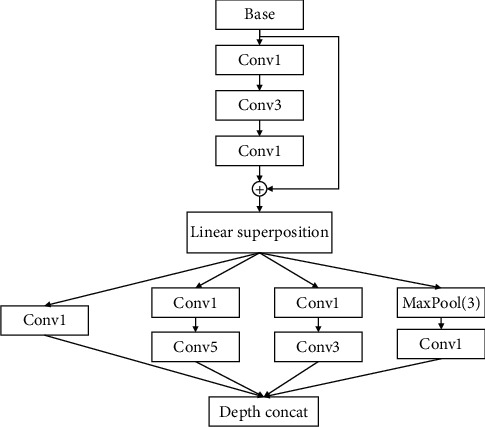
GFBE structure.

Different convolutional layers of CNN can extract different features of the target. The shallow convolutional layer extracts the features of the target such as texture and contour, while the deep convolutional layer extracts the abstract features of the target and contains richer semantic information. However, with the deepening of the network layers, there will be problems such as information loss, gradient disappearance, and degradation. The location distribution of TFI information for different classes of radar signals is different, so this paper designed a global feature balance extraction module (GFBE), as shown in Figure 1. In Figure 1, “Conv1,” “Conv3,” and “Conv5” represent 1 × 1, 3 × 3, and 5 × 5 convolution kernels, respectively, and “Maxpool (3)” represents a 3 × 3 pooling layer with a stride of 1. The module contains multiple sizes of convolution kernels. The short skip connection layer of the module is composed of two “Conv1” and “Conv3”. Through the short skip connection, it can prevent information loss, increase the network depth, and solve the problem of network degradation to a certain extent. The first Conv1 is used to reduce the dimension, and the second Conv1 is used to increase the dimension. The main purpose is to reduce the number of parameters and increase the nonlinear learning ability of the network. The next is the parallel convolution structure and point convolution layer, which contains convolution kernels of various sizes: “Conv5,” “Conv3,” “Conv1” and 3 × 3 MaxPool. For TFI of different radar signals, larger convolution kernel are used for images with more dispersed information distribution, while a smaller convolution kernel is used for images with more local information distribution, which can ensure balanced extraction of image features.

### 2.2. IIF-Nets Structures

Based on the GFBE module, 3 IIF-Net deep CNN structures are proposed: IIF-Net56, IIF-Net107, and IIF-Net Net158. In these networks, a GFBE structure has 5 layers, where a “Conv” is a composite structure containing “convolution,” “batch standardization,” and “activation function”. The network structure is shown in Table 1.

Radar signal recognition technology requires high real-time performance, and recognition must be made immediately when the signal is captured. The network is required to have less parameters and low calculation cost to reduce the consumption of hardware, so the global average pooling (GAP) [19] is used as the classifier of IIF-Net. This classification method does not require a fully connected layer, which can greatly reduce the number of parameters and can avoid overfitting under certain conditions.

### 2.3. Network Complexity

When different classifiers are used to identify 8 classes of radar signals, the network parameters and calculations are different. Suppose the size of the output feature map of the last layer is *H* × *W* × *D*, when using three fully connected layers, the number of parameters in the classifier is 16,818,184+4096 × *H* × *W* × *D*. When a single-layer fully connected layer is used, the parameters in the classifier are *H* × *W* × *D* × 8+8. When using GAP, since the pooling layer has no parameters, the number of parameters can be further reduced to *D* × 8+8.

**Figure 2:**
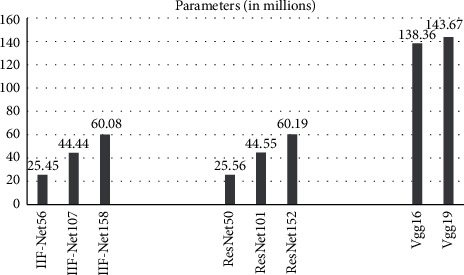
Parameters.

**Figure 3:**
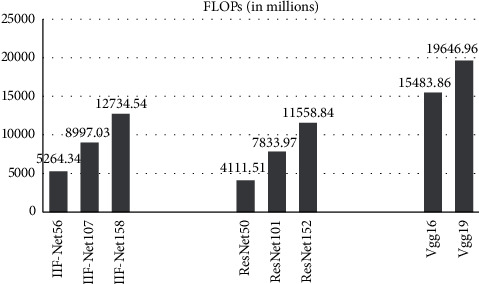
FLOPs.

The number of parameters for different networks is shown in Figure 2, and the number of calculation is shown in Figure 3.

It can be seen from Figure 2 that IIF-Net slowly increases the parameter amount with the increase of the network depth, and the network depth has little effect on the parameter amount. The VGG16 network has only 16 layers, but the amount of parameters is 5.44 times that of IIF-Net56, 3.11 times that of IIF-Net107, and 2.30 times that of IIF-Net158. IIF-Net has 6 more layers than ResNet, but the number of parameters is reduced by about 110,000. The radar system requires high real-time performance, but the small equipment, such as bombs, has insufficient memory, and its hardware is hard to support too many parameter quantities. IIF-Net is relatively small in parameter quantity, which is a kind of a better choice.

According to Figure 3, the calculation of the VGG network is very huge. The floating-point operations per second (FLOPs) of VGG16 is as high as 15.583 billion, which is 2.94 times that of the 56-layer IIF-Net. Network structure and network depth have a great impact on the amount of computation. IIF-Net is deeper than ResNet, so the amount of calculation is increased. The number of layers of IIF-Net107 is 1.80 times that of IIF-Net56, so the amount of calculation is 1.71 times that of IIF-Net56. The amount of IIF-Net158 is 2.42 times that of Net56, which is very huge. Therefore, when the difference in the signal recognition rate is not large, IIF-Net56 has the highest cost performance.

## 3. Experimental Results

### 3.1. Dataset

The dataset is generated by USRP N210, USRP-LW N210 simulating the process of real radar signal transmission and reception. The generated signal is transformed by CWD to obtain TFI. Unlike SAR images [20] in radar target recognition and high-resolution radar target images [21], TFI is a digital image with low image information loss, which is convenient for computer processing and analysis.

There are many methods of time-frequency analysis, including short-time Fourier transform (STFT), continuous wavelet transform (CWT), bilinear models including Wigner–ville distribution, pseudosmooth (WVD), CWD, adaptive parameter models (such as the ARMA model, time-frequency rearrangement model (RS), and synchronous extraction model SET). But, they have some shortcomings. For example, the time-frequency resolution of STFT and CWT is insufficient. The effect of WVD on multicomponent signal interference is poor. The RS complexity is too high; SST and SET are very advantageous for instantaneous frequency extraction and signal reconstruction, but the signal energy is too compressed, resulting in only one line at the frequency point. In this paper, high definition CWD transform is adopted, and an appropriate mask function is selected to avoid the cross-term problem, which improves the recognition performance of the radar signal.

The Choi–Williams distribution function is one of a series of Cohen's class distribution functions. The distribution uses an exponential core function to filter out cross terms. The core function of the Choi–Williams distribution does not increase with the increase of *μ* and *τ*, so it can filter out the cross terms with different frequencies and time centers.

(1) $$\begin{array}{c} C_{x}\left(t,f\right)=\int_{-\infty }^{\infty }\int_{-\infty }^{\infty }A_{x}\left(\mu ,\tau \right)\varphi \left(\mu ,\tau \right)\exp\left(j2\pi \left(\mu t-\tau f\right)\right)\text{d}\mu \text{d}\tau , \end{array}$$

where *A*_*x*_(*μ*, *τ*)=∫_−*∞*_^*∞*^*x*((*t*+*τ*)/2)*x*^*∗*^((*t* − *τ*)/2)*e*^−*j*2*πtμ*^d*t* is the fuzzy function, *μ* and *τ* are, respectively, the frequency offset and delay, and *x*(*t*) is the received signal.

The core function *φ*(*μ*, *τ*)=exp[−*α*(*μτ*)^2^] is a Gaussian function, where *α*  is an adjustable parameter.

**Figure 4:**
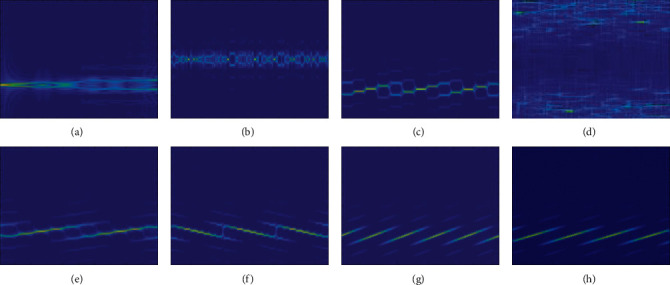
TFI of various radar signals. (a) Barker, (b) Frank, (c) chaotic, (d) OFDM, (e) P1, (f) P2, (g) P3, and (h) P4.

In the radar signal dataset, there are 8 types of signals. Each class of signal generates 2592 TFIs, and the SNR is −10∼6 dB. Each class of signal has a total of 20,736 samples, and every 2 dB contains 288 samples.  Figure 4 shows the TFI of the signal after passing through CWD.

It can be seen from the images that the distribution of different signal information is different: the distribution of chaotic code information is relatively concentrated, the distribution of OFDM signal information is relatively scattered, and the information distributions of P1–P4, Barker, and Frank are below the center, with irregular signal characteristics.

### 3.2. Preprocessing

In the experiments, we downsample the samples of the training set and the test set to a fixed resolution of 224 × 224 and, then, expand the data: randomly flip the image horizontally, randomly flip vertically, and randomly rotate 90°. The data set is expanded by 3 times to prevent the network from overfitting.

In order to maintain the unity of the experiments, the experiments are conducted on the same platform. The platform of signal generation is shown in Table 2.

During the experiment, the parameters were set up, the learning rate is 0.001, the momentum is 0.9, the weight decay is 5*e* − 4, and the batch size is 10. The experimental platform configuration is shown in Table 3.

### 3.3. Experimental Results

In order to make the radar signal recognition more authentic and simulate the interference of a complex external environment, noises with an SNR of −10∼6 dB are added to the signal. The real radar signal transmission and reception process is simulated by USRP N210 and USRP-LW N210. The generated signals are transformed by CWD to obtain TFI for radar signal identification. Under the same training set and test set, we use different depths of IIF-Net to identify radar signals under different SNRs. The experimental results are shown in Table 4.

According to Table 4, the signal recognition rate of IIF-Net56 is 99.36% and in the case of SNR is −4 dB. When the SNR is −10 dB, the noise causes a lot of interference, but the recognition rate is still higher than 92%. The results indicate that the IIF-Net networks are robust. The recognition rate of IIF-Net56 is about 1% lower than that of the other 2 networks. It shows that, with the deepening of network depth, there is no obvious difference in the extraction of signal features. The parameter amount of IIF-Net158 and IIF-Net107 is 2.36 times and 1.75 times of that of IIF-Net56, and the calculation amount is 2.42 times and 1.71 times of that of IIF-Net56. Based on the experimental results, we found that IIF-Net158 had the best recognition performance, but the network parameters and calculation amount increased greatly. Therefore, based on the abovementioned analysis, IIF-Net56 has the highest cost-performance ratio.

Under the same training set and test set, we also compare IIF-Net56 with other networks. Experimental results of other CNN networks are shown in Table 5.

According to Table 5, various classic CNNs have a good recognition rate for radar signals when the SNR is above 0 dB. However, when the SNR is between −10 dB and 0 dB, IIF-Net has the highest recognition performance. Compared with IIF-Net, the signal recognition rate of VGG-Net is about 6% lower than that of IIF-Net. Because of VGG-Net's shallow network, it cannot fully extract the features of the image, resulting in low signal recognition rate. Moreover, VGG-Net has too large parameters and calculation and requires too much hardware equipment and more calculation time. Therefore, VGG-Net is not suitable for the radar electronic countermeasure field which needs high real-time performance.

The signal recognition rate of ResNet is close to IIF-Net, which is about 2% lower. Because ResNet uses short skip connection, it can deepen the network and solve the problem of “network degradation” to a certain extent. It can also prevent information loss during network transmission. However, the distribution of TFI feature information of a radar signal is irregular, and ResNet mostly uses small convolution kernel of 3 × 3, which has good recognition effect for images with concentrated information distribution and has low recognition effect for TFI features of radar signal. The GFBE module proposed in this paper solves this problem to a certain extent. For images with different information distribution, it can extract image features in a global and balanced way, improve signal recognition rate, and enhance generalization.

We further compare IIF-Net56 with other radar signal recognition methods, and the results are shown in Table 6.

According to Table 6, the signal recognition rate of the DQN network at −6 dB is higher than that of IIF-Net56, which is 1.05% higher, but at −10 dB, the recognition rate is much lower than that of IIF-Net56, which is reduced by 4.81%. This indicates that high-intensity noise has little influence on IIF-Net, and IIF-Net can still fully extract image information, obtain high signal recognition rate, and have good robustness. It can also be seen from the table that when the SNR is above −6 dB, the signal recognition rate obtained by I-CNN has little difference from that of IIF-Net, and both of them have good recognition effect. When the SNR is −10 dB and −8 dB, the signal recognition rate of IIF-Net is much higher than that of I-CNN, which shows that IIF-Net has strong anti-interference ability and can extract image features in a balanced and sufficient way. Fusion Image uses transfer learning and a cascaded automatic encoder based on self-learning to extract the effective information of the fused image, thereby ensuring the recognition performance. Meanwhile, Fusion Image adopts multifeature Fusion algorithm to fuse features, which reduces redundant information of features, but its recognition rate is 1.03% lower than that of IIF-Net56 at −6 dB. FCBF-AdaBoost and Entropy are traditional image classification methods, which are mostly designed for certain classes of image features. Their recognition rates are relatively poor in multitask and low SNR environments.

Under the same training set and test set, the recognition rates of IIF-Nets proposed in this paper under different SNRs are shown in Table 7.

It can be seen from Table 7 that, under the environment of low SNR (−10 dB), 3 IIF-Net networks have little difference in the recognition effect of different radar signals. The deepening of the network depth has a significant effect on the recognition rate of various radar signals. The influence range of network depth on the recognition rate of various radar signals is between 1% and 2%. This indicates that when the network depth reaches a certain degree, the signal feature information can be fully extracted. Further deepening of the network has little impact on the recognition effect of signals, but the recognition effects of different classes of radar signals under the same network are greatly different. Among them, Barker has the best recognition effect, over 97%. chaotic, Frank, OFDM, P2, and P3 receive the next highest recognition rates, with accuracy rates of over 94 percent, while P1 and P4 have relatively poor recognition effects, at about 80 percent. According to the TFI of the radar signal, P1 and P4 are very similar. Under the environment of −10 dB, the energy of noise is much greater than that of the signal, and the information features of the signal are drowned by the noise, which makes P1 and P4 more similar and greatly increases the difficulty of identification. However, IIF-Net56 has a comprehensive recognition rate of 92.36% under −10 dB, and its recognition performance is higher than that of other methods.

The IIF-Net proposed in this paper can extract information globally for images with irregular information distribution, which has a good recognition effect. Other traditional methods are mostly designed for specific classes of images. When the image changes greatly, their recognition effects are poor. The artificially designed feature extraction algorithm is also relatively complex, and its generalization performance is low. Compared with other CNNs, IIF-Net still has a recognition rate of 92.36% under −10 dB, which is higher than that of those other CNNs.

### 3.4. Experiments Analysis

This paper proposes 3 IIF-Net structures, namely, IIF-Net56, IIF-Net107, and IIF-Net158. According to the experimental results, their signal recognition rates are above 99.74% when the SNR is higher than −2 dB. At −10 dB, the recognition rates are as high as 92.36%. When deepening the networks, the differences between the recognition rates of the three networks are within 1%, but the parameters and calculations have increased significantly. Therefore, IIF-Net56 has the best overall performance.

The information characteristic distribution of the radar TFI signal is irregular. Therefore, the distribution characteristics and irregularity of image information should be taken into account when extracting image features. A parallel convolutional layer can be used to extract different types of image information. The network depth should be kept moderate. It is difficult to fully extract image features when the network is too shallow, but the recognition rate cannot be significantly improved when the network is too deep. If the network is too deep, the degradation problem may occur, and the amount of parameters and calculation will increase greatly. To a certain extent, the problem of network degradation can be solved by using a short skip connection mode, while the integrity of image information can be maintained. The classifier can choose GAP to reduce the number of network parameters and calculations. The GFBE module includes Conv1, Conv3, Conv5, and MaxPoo(3) to deepen the network through short skip connection to prevent the loss of image information and uses Conv3, Conv5, and the MaxPool(3) parallel convolutional layer to extract global information. At the same time, it controls the dimensions of the network through Conv1 and improves the nonlinear learning ability of the network.

## 4. Conclusions

In this paper, USRP N210 and USRP-LW N210 are used to simulate the transmitting and receiving process of radar signals to generate near-real radar signals. Then, CWD is used to get the radar TFI. According to the irregular information distribution characteristics of radar signal TFI, we designed a GFBE module. Based on this module, three network structures, IIF-Net56, IIF-Net107, and IIF-Net158, are proposed. Through analysis, we conclude that IIF-Net56 has the best comprehensive performance. The network has a recognition rate of 92.36% at a low SNR of −10 dB. GAP is added into the network, and the number of parameters and calculation amount are relatively less, which reduces the requirement for hardware equipment. IIF-Net56 uses a GAP layer to reduce the amount of parameters and calculation and reduces the requirements of hardware equipment. Therefore, the network proposed in this paper has a good application prospect in the field of high real-time radar electronic countermeasures. In the field of radar electronic countermeasures, transmitting jamming signals for electronic countermeasures is a common method. In the future, we will do further research on radar jamming signal recognition.

## Figures and Tables

**Table 1:** IIF-Net configuration.

IIF-Net56		IIF-Net107		IIF-Net158	
Conv7-64, stride: 2, padding: 3 × 3 Maxpool, stride: 2, padding: 1					
Conv1-64 Conv3-64 Conv1-256	×2	Conv1-64 Conv3-64 Conv1-256	×2	Conv1-64 Conv3-64 Conv1-256	×2

GBFE-256					
Conv1-128 Conv3-128 Conv1-512	×3	Conv1-128 Conv3-128 Conv1-512	×3	Conv1-128 Conv3-128 Conv1-512	×7

GBFE-512					
Conv1-256 Conv3-256 Conv1-1024	×5	Conv1-256 Conv3-256 Conv1-1024	×22	Conv1-256 Conv3-256 Conv1-1024	×35

GBFE-1024					
Conv1-512 Conv3-512 Conv1-2048	×3	Conv1-512 Conv3-512 Conv1-2048	×3	Conv1-512 Conv3-512 Conv1-2048	×3

GAP					
Classifier, Soft-max					

**Table 2:** Signal generation platform configuration.

Parameter	USRP N210/USRP-LW N210
REF IN	15 dBm
PPS IN	5 V
Power	6 V, 3 A
ADC sampling rate	100 MS/s
DAC sampling rate	400 MS/s
LO accuracy	2.5 ppm

**Table 3:** Experimental platform configuration.

Attributes	Configuration information
Operating system	Ubuntu 14.04.5 LTS
CPU	Intel (R) Xeon (R) CPU E5-2670 v3 @ 2.30 GHz
GPU	GeForce GTX TITAN X
CUDNN	CUDNN 6.0.21
CUDA	CUDA 8.0.61
Frame	PyTorch

**Table 4:** IIF-Net recognition accuracies at different depths (%).

SNR (dB)	IIF-Net56	IIF-Net107	IIF-Net158
−10	92.36	92.54	92.85
−8	94.55	95.56	95.64
−6	96.53	96.73	97.52
−4	99.36	99.48	99.53
−2	99.74	100	100
0	100	100	100
2	100	100	100
4	100	100	100
6	100	100	100

**Table 5:** Recognition accuracy rates of other CNNs (%).

SNR (dB)	ResNet50	ResNet101	ResNet152	VGG16	VGG19	IIF-Net56
−10	90.49	90.85	91.24	86.85	88.59	92.36
−8	92.68	93.79	94.46	89.26	90.27	94.55
−6	94.65	95.15	96.31	92.57	94.16	96.53
−4	97.47	97.83	98.52	95.61	96.54	99.36
−2	98.87	99.26	99.49	98.42	99.62	99.74
0	99.51	100	100	99.53	99.75	100
2	100	100	100	100	100	100
4	100	100	100	100	100	100
6	100	100	100	100	100	100

**Table 6:** Recognition accuracy rate of other methods (%).

Method	−10	−8	−6	−4	−2	0	2	4	6
DQN [8]	87.55	—	97.58	—	—	—	100	100	100
Entropy [1]	66.50	—	—	—	—	100	—	—	—
FCBF-AdaBoost [6]	—	—	—	—	—	94.46	96.86	98.75	98.52
Fusion Image [22]	—	—	95.50	—	—	—	—	—	—
I–CNN [23]	55	80	96.10	—	—	100	100	100	100
IIF-Net56	92.36	94.55	96.53	99.36	99.74	100	100	100	100

**Table 7:** Recognition accuracy of the same signal in different networks (−10 dB) (%).

Signal	IIF-Net56	IIF-Net107	IIF-Net158
Barker	100.00	97.22	100.00
Chaotic	96.56	100.00	97.35
Frank	95.83	98.61	96.26
OFDM	96.54	100.00	98.85
P1	81.67	79.17	80.37
P2	95.44	94.44	94.68
P3	94.72	97.22	95.84
P4	80.52	80.56	81.41

## Data Availability

The dataset in the paper can be obtained by contacting Huiqiang Zhang (hqzhang9013@163.com).
